# A correlativity study of plasma APL1β28 and clusterin levels with MMSE/MoCA/CASI in aMCI patients

**DOI:** 10.1038/srep15546

**Published:** 2015-10-27

**Authors:** Ying Meng, Huiying Li, Rui Hua, Huali Wang, Jian Lu, Xin Yu, Chen Zhang

**Affiliations:** 1 Dementia Care & Research Center, Peking University Institute of Mental Health; Beijing Municipal Key Laboratory for Translational Research on Diagnosis and Treatment of Dementia, National Clinical Research Center for Mental Disorders (Peking University Sixth Hospital), Peking University; 2 State Key Laboratory of Membrane Biology, School of Life Sciences; PKU-IDG/McGovern Institute for Brain Research, Peking University, Beijing 100871, China

## Abstract

Amnestic mild cognitive impairment (aMCI) is a sub-clinical condition characterized by memory deficits that are not severe enough to affect daily functioning. Here we investigated two potential biomarkers found in the cerebrospinal fluid of AD patients, APLP1-derived Aβ-like peptides 28 (APL1β28) and clusterin plasma levels, in terms of their relationship to cognitive function, as reflected in the Mini-Mental State Examination (MMSE), the Montreal Cognitive Assessment (MoCA) and the Cognitive Assessment Screening Instrument (CASI) in aMCI patients. Forty-seven aMCI patients and thirty-five age- and gender-matched healthy adult controls were recruited for this study. Using the ELISA method, we found that the mean concentrations of both APL1β28 and clusterin were not significantly different between the control and aMCI groups. The APL1β28 levels were positively correlated with clusterin and that both were negatively correlated with the MMSE scores of the aMCI patients. Clusterin levels were negatively correlated with the MoCA and CASI scores of the aMCI patients. Using multivariate analysis, the correlation between clusterin and MMSE/MoCA/CASI was independent of other AD risk factors including age, education, sex, body mass index and ApoE genotype. The data presented here demonstrate that plasma clusterin levels reflect cognitive function in aMCI patients.

Amnestic mild cognitive impairment (aMCI), which affects 5–6% of people older than 65, is a syndrome attributed to people with noticeable impairments in memory function but whose other cognitive functions are normal^1^,^2^,^3^,^4^,^5^,^6^,^7^,^8^. Similar to other forms of mild cognitive impairment (MCI) such as non-memory (nmMCI) and multi-domain (mMCI) subtypes, aMCI patients retain normal daily activities. It is estimated that, annually, 7% of MCI cases convert to Alzheimer’s disease (AD) and other dementia subtypes^9^,^10^, chronic diseases in which the progression of brain changes and cognitive impairments usually appear 10–20 years before the clinical diagnosis. Similar to AD and other forms of MCI, the exact causes of aMCI are not clearly understood, though age and family history are shown to be risk factors. There currently is no cure for dementia, including AD, and most clinical trials targeting AD patients in the dementia phases have not shown promising effects. As a result, there have been mounting efforts to identify disease-related changes in the pre-dementia phase, such as in aMCI, for early diagnosis and intervention.

At this time, the diagnosis of aMCI depends mainly on a comprehensive medical evaluation that includes neuropsychological testing and assessment of the patient’s history^11^,^12^,^13^,^14^,^15^,^16^,^17^,^18^,^19^. Biomarkers, including imaging and biochemical substances in the body fluids, assist the disease diagnosis and allow for the monitoring of the pathogenesis. For example, magnetic resonance imaging (MRI) has shown atrophy in the medial temporal lobe, including the hippocampus and entorhinal cortex, in MCI (including aMCI) and AD populations^5^,^20^,^21^,^22^,^23^, and carbon 11-labeled PiB (Pittsburgh compound B) PET has demonstrated increased brain amyloid burden in aMCI and AD patients^23^,^24^,^25^,^26^,^27^,^28^,^29^,^30^,^31^,^32^,^33^. In addition to imaging methods^13^,^32^,^33^,^34^,^35^, body fluid biomarkers are attracting more attention due to their direct relevance to the biology of dementia^36^,^37^,^38^,^39^. Low concentrations of Aβ42 and high concentrations of total and phosphorylated tau have been detected in the CSF of MCI and AD populations from mono-center and multi-center longitudinal studies^40^,^41^,^42^,^43^,^44^,^45^,^46^,^47^,^48^,^49^,^50^. Diagnostic sensitivity to CSF Aβ42 and Tau in MCI patients due to AD is about 50–90%, and the specificity is 80–100%^40^,^41^,^42^,^43^,^44^,^50^. Moreover, compared to MRI and CSF biomarkers, the use of plasma biomarkers as a diagnostic tool is relatively low-cost and non-invasive. Based on CSF biomarker studies and on MRI, brain atrophy and Aβ deposits in the patient’s brain suggest that Aβ and proteins implicated in Aβ metabolism are potential monitors for AD and MCI pathogenesis^37^,^38^,^51^,^52^,^53^,^54^,^55^. Generated by similar β- and γ-cleavages on amyloid-like protein 1 (APLP1, another member of the amyloid precursor protein [APP] gene family), which also includes APLP2 and APP), APL1β28 was recently reported to be a surrogate biomarker for AD with the use of mass spectroscopy^56^. Similar to APP, which produces the amyloid beta species (Aβ40, Aβ42), a component of senile plaque^57^,^58^,^59^, APLP1 undergoes α-, β- and γ-secretase; unlike APP, however, no Aβ peptide is generated due to the lack of an Aβ sequence in the gene^60^,^61^,^62^,^63^. The APL1β28 level is higher in the CSF of patients with MCI and familial Alzheimer’s disease (FAD), as well as that in patients with sporadic AD^56^,^64^. Besides the Aβ-related APP gene family, proteins implicated in APP metabolism are also potential targets for biomarkers. Clusterin, the third-strongest genetic risk factor for late-onset Alzheimer’s disease (LOAD), is implicated in the clearance process of Aβ accumulation and is associated with the rate of cognitive decline^65^,^66^,^67^,^68^,^69^,^70^. Clusterin, also called apolipoprotein J, is a disulfide-linked heterodimeric protein whose SNPs, according to genome-wide association studies, are linked to AD^71^,^72^. Moreover, increased levels of CSF clusterin have been found to be connected to entorhinal atrophy in AD and MCI patients with high Aβ deposition. Plasma clusterin concentration has been reported to be associated with Mini-Mental State Examination (MMSE) scores in combined MCI/AD cohorts^73^. However, the relationship between the plasma concentrations of APL1β28 and clusterin, two potential biomarkers implicated in APP processing and metabolism, has not been examined in the aMCI population.

Furthermore, there are a number of risk factors that are strongly linked with SAD. For example, the strongest genetic risk factor in the pathogenesis for LOAD and MCI is considered to be ApoE polymorphisms^74^,^75^,^76^,^77^. Epidemiological studies have shown that age is the most dominant risk factor for the development of AD and MCI^78^,^79^,^80^; a high level of education has also been connected to a lower incidence of AD^81^,^82^. Body mass index (BMI) has been shown to be associated with the CSF biomarkers of amyloid and tau in MCI patients^83^. Thus, whether these factors, combined with plasma APL1β28 and clusterin levels, might have a synergic effect remains to be determined. In the current study, we focused our analysis on aMCI populations and aimed to investigate whether plasma levels of APL1β28 and clusterin are correlated with cognitive status in the aMCI population and whether these correlations have relationships with other risk factors associated with aMCI and AD.

## Results

**Figure 1:**
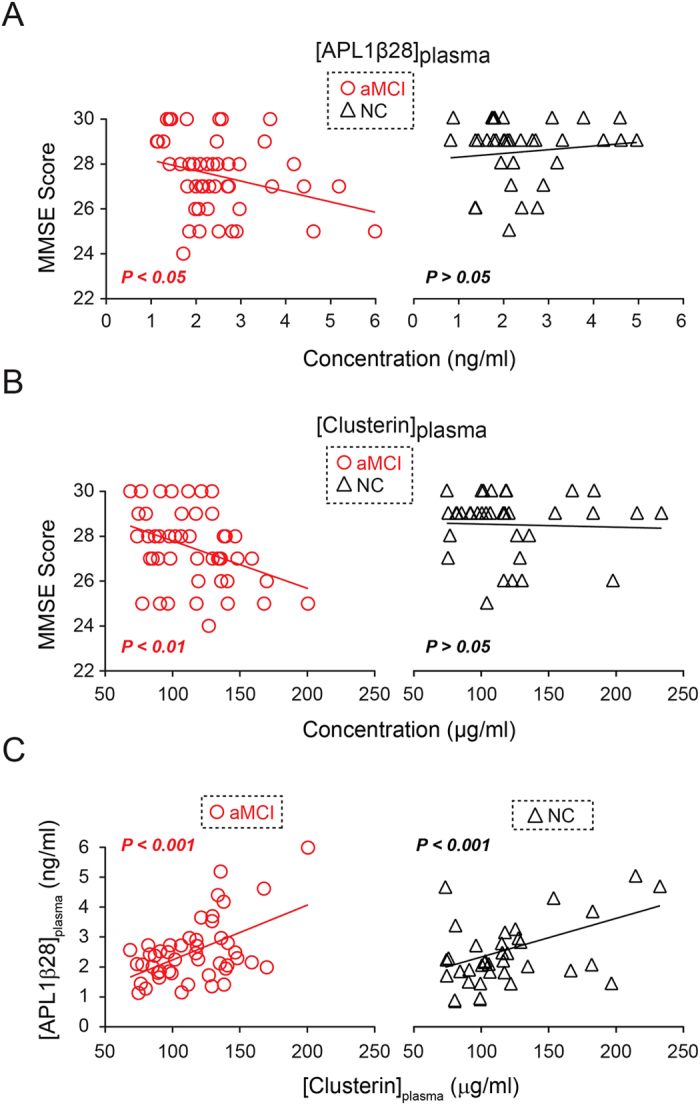
Statistical correlations between MMSE scores and plasma concentrations of APL1β28 and clusterin. Scatter plots show correlations in all subjects and separately in the individual diagnostic groups. Solid lines indicate linear regression. (**A**) MMSE scores in the aMCI group (red circle) negatively correlate with APL1β28 concentrations in plasma. (**B**) MMSE scores in the aMCI group (red circle) negatively correlate with clusterin concentrations in plasma. (**C**) Plasma concentrations of APL1β28 and clusterin show strong correlations in both the aMCI (red circle) and NC (black triangle) groups. Note: Statistical comparisons were made using the student’s t-test.

**Figure 2:**
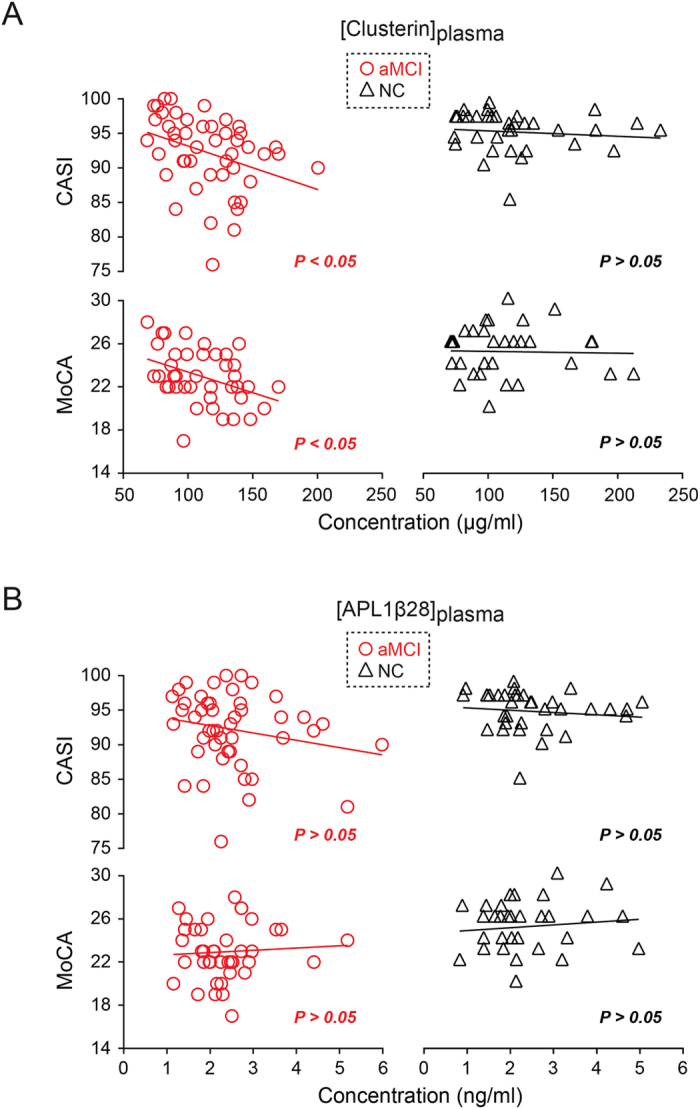
Statistical correlations between CASI and MoCA scores and plasma concentrations of APL1β28 and clusterin. (**A**) CASI and MoCA scores in the aMCI group (red circle) negatively correlate with clusterin concentrations in plasma. (**B**) Plasma concentrations of APL1β28 do not correlate with CASI and MoCA scores in the aMCI or NC groups.

Forty-seven aMCI patients and thirty-five age-matched healthy controls were recruited for our study. All patients were of Chinese Han ethnicity. Detailed demographic data of all aMCI and NC subjects are presented in Table 1. The enzyme-linked immunosorbent assay (ELISA) was performed to determine the absolute concentrations of APL1β28 and clusterin in plasma ([APL1β28]_plasma_ and [clusterin]_plasma_, respectively), and we adopted ELISA methods that have been applied and validated previously^73^. The standard curves for APL1β28 (Supplementary Figure 1) and clusterin (Supplementary Figure 2) show reliable and reproducible measurements. Our data indicate that the mean [APL1β28]_plasma_ and [clusterin]_plasma_ levels were not significantly different between the aMCI and control groups (APL1β28: aMCI group, 2.51 ± 0.15 ng/ml, NC group, 2.41 ± 0.17 ng/ml, p = 0.66; clusterin: aMCI group, 115.27 ± 4.30 μg/ml, NC group, 120.32 ± 6.87 μg/ml, p = 0.52). We then performed a one-sample Kolmogorov-Smirnov test on plasma APL1β and clusterin concentrations, and on the MMSE, MoCA, CASI scores in the aMCI and NC groups. All data followed a normal distribution except for the MMSE scores in the NC group (Table S1). We thus adopted Spearman’s rank correlation analysis for all the correlations with MMSE in the NC group and Pearson’s correlation for all other correlation analyses. We tested the correlations between plasma APL1β28 and clusterin levels using scores from the MMSE, which is the most widely used screening instrument for cognitive deficits. As shown in Fig. 1A, the analysis of correlations between [APL1β28]_plasma_ and the MMSE scores in the aMCI subjects demonstrated negative correlations (r = −0.293, p = 0.046), while no significant relationship was observed in the NC group (r = −0.028, p = 0.871). A similar result was obtained for plasma clusterin concentration (Fig. 1B, aMCI group: r = −0.377, p = 0.009; NC group: r = −0.096, p = 0.584). These results indicate that aMCI patients with higher APL1β28 or clusterin levels tend to have lower MMSE scores. Furthermore, the correlation between [APL1β28]_plasma_ and [clusterin]_plasma_ was very strong in both the aMCI and NC populations (Fig. 1C, aMCI group: r = 0.518, p < 1.89 × 10^−4^; NC group: r = 0.445, p = 0.007). Despite the MMSE’s popularity, the test is less sensitive to milder forms of cognitive impairment and can be affected by factors such as race, education, and language ability^84^,^85^. Thus, other dementia screening tools have been developed and are well known to allow a more comprehensive understanding of other cognitive domains. The CASI, introduced in 1994, features a scale of 100 points^86^ and combines the MMSE and the Hasegawa Dementia Rating Scale (HDRS)^87^. Featuring more detailed assessments and broader scoring ranges, the CASI is considered more useful for determining the severity of dementia. In our study, [clusterin]_plasma_ was also found to be negatively correlated with CASI scores in the aMCI subjects (Fig. 2A, r = −0.346, p = 0.017), while [APL1β28]_plasma_ was not found to be correlated (Fig. 2B, r = −0.206, p = 0.164). Additionally, in the NC group, the CASI scores were not correlated with [clusterin]_plasma_ or with [APL1β28]_plasma_ (Fig. 2A, r = −0.115, p = 0.512; Fig. 2B, r = −0.125, p = 0.474).

The MoCA, introduced by Nasreddine *et al.*^88^ in 2005, is designed specifically to be used for patients with MCI or with mild AD. The [clusterin]_plasma_ of aMCI patients, but not that of the controls, was negatively correlated with the MoCA scores (Fig. 2A, aMCI group: r = −0.392, p = 0.012; NC group: r = −0.031, p = 0.870). The MoCA scores in the aMCI and NC groups were not correlated with [APL1β28]_plasma_ (Fig. 2B, left panel, r = 0.068, p = 0.677; Fig. 2B, right panel, r = 0.114, p = 0.542).

Age is unquestionably the strongest risk factor for the development of AD and MCI^78^,^79^,^80^. Thus, we performed multivariate analysis to evaluate whether age is a factor in the correlations between APL1β28 or clusterin levels in plasma and MMSE/MoCA/CASI scores. Our data show that the correlations of clusterin or APL1β28levels with MMSE scores were independent of age (Table 2). After adjusting for age, the correlations of clusterin levels with MoCA or CASI scores remained significant, and those of APL1β28 remained not significant. Next, we tested whether education level or sex might affect these correlations. As summarized in Table 2, after adjusting for education and sex, clusterin levels correlate with MMSE, MoCA and CASI significantly; however, only the correlation efficiency of APL1β28 with MMSE showed a trend toward significance (p = 0.055 and 0.059, respectively). The correlation of APL1β28 with MoCA and CASI remained not significant after adjusting for education and sex.

**Figure 3:**
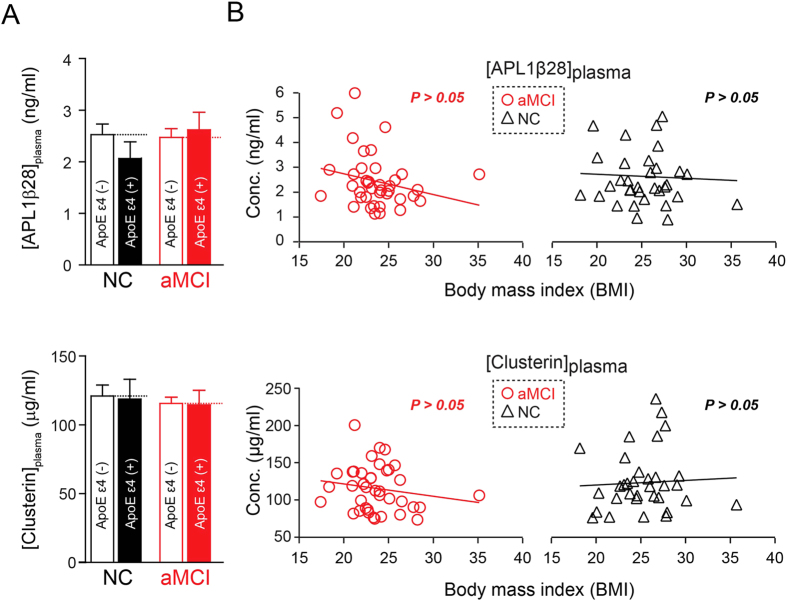
Statistical correlations between ApoE genotype, BMI and plasma concentrations of APL1β28 and clusterin in the aMCI or NC groups. (**A**) No significant changes are observed in the APL1β28 and clusterin levels of the aMCI and NC groups. All summary graphs show a mean ± SEM. (**B**) Plasma concentrations of APL1β28 and clusterin do not correlate with BMI in the aMCI or NC groups.

The strongest genetic risk factor in the pathogenesis for AD and MCI is ApoE polymorphisms^74^,^75^,^76^,^77^. To determine whether there is interaction between the ApoE genotype and plasma APL1β28 and clusterin levels, we compared the average levels of these two proteins in the blood of ApoE ε4-carriers and non-carriers. We used Sanger’s sequencing to examine the ApoE polymorphisms in aMCI and control groups and found that 27.7% of the aMCI patients (13 out of 47) and 25.7% of the control patients (9 out of 35) carried a single copy of ApoE ε4 allele, while no subject carrying two copies was identified in either the aMCI or control groups. Our results show that the plasma levels of APL1β28 and clusterin are not affected by the presence/absence of ApoE ε4 allele (Fig. 3A). Furthermore, we tested the effects of ApoE genotype on the correlations between these two proteins with cognitive scores. The data showed that ApoE genotype has no effect on clusterin’s correlation with MMSE, MoCA and CASI scores, but that it increased the correlation efficiency of APL1β28 with the MMSE score from 0.046 to 0.053. To determine whether BMI is associated with plasma APL1β28 and clusterin levels, we plotted the concentration versus the BMI measured at the time of clinical examination. As shown in Fig. 3B, there is no association between APL1β28 and clusterin levels and BMI values in either aMCI or NC populations (APL1β28-aMCI group: r = −0.238, p = 0.144; NC group: r = 0.010, p = 0.954; clusterin-aMCI group: r = −0.168, p = 0.307; NC group: r = 0.165, p = 0.350).

## Discussion

In this study, we found that the MMSE scores reflecting cognitive ability were negatively correlated with plasma APL1β28 and clusterin levels in aMCI patients but not in the levels of healthy controls. In addition, the plasma clusterin level was also correlated with CASI and MoCA scores in the aMCI population. These results are consistent with findings showing that APL1β28 and clusterin levels in CSF are altered in the MCI, PS1-FAD, and sporadic AD patients^56^,^89^,^90^. Since the distribution of MMSE scores in the NC groups were within a narrow range and did not follow a normal distribution, we performed parametric analysis (Pearson’s correlation) and non-parametric analysis (Spearman’s rank correlation) and found no correlations in the NC groups.

The correlation between clusterin and MMSE, MoCA or CASI seems specific, since it is independent of age, education level, BMI, and ApoE polymorphisms, as revealed by multivariate analysis. Furthermore, plasma clusterin and APL1β28 levels showed a very strong correlation in both aMCI and control subjects, a finding that is consistent with the notion that they are both involved in the processing of APP. These observations add to the existing evidence that suggests a convergent role of APP dysfunction in the decline of cognitive ability. This finding is consistent with previous reports showing that clusterin interacts with Aβ to cause AD pathology. For instance, clusterin concentrations in CSF show a significant interaction with CSF Aβ_1-42_ on the atrophy rate of the entorhinal cortex in AD and MCI patients, as well as that in healthy controls^89^. Moreover, plasma clusterin levels have been found to be an indicator for the rate of cognitive decline and brain atrophy in AD patients^73^,^89^,^91^,^92^. Although Aβ_25-35_ treatment of primary neurons elevates intracellular clusterin levels and reduces extracellular-secreted clusterin specifically, treatment with other stressors, including MG132 (the proteasome inhibitor), and ultraviolet irradiation causes cell death without significant changes in clusterin levels. Furthermore, the inactivation of clusterin expression in neurons reduces Aβ-mediated neurotoxicity^93^. In transgenic mice carrying FAD mutations (PS1M146V, APP_Swe,_ and tauP301L), clusterin in the serum has been reduced by about 30%; this reduction can be fully rescued by treating the mice with coenzyme Q10^94^.

When compared to those of clusterin, the correlations of APL1β28 with cognitive scores are rather weak, reaching a significant level only when correlated with MMSE (p = 0.046) but not when correlated with MoCA or CASI. Furthermore, after adjustment for sex, education level or ApoE genotype, the correlation remains not significant (p = 0.05–0.06). We speculate that there might be two possibilities for this: the first is that the weak correlation between APL1β28 with MMSE is because APL1β28 strongly correlates with clusterin, and clusterin in turn strongly correlates with MMSE. That is to say, the [APL1β28]_plasma_-MMSE correlation is a consequence of clusterin’s effect on the cognitive ability of aMCI subjects. The second possibility is that the plasma levels of APL1β28 might not accurately reflect the secretase activity in the brain. Therefore, further studies are required to determine whether APL1β share identical cleavage processes with Aβ and whether the levels of [APL1β28]_plasma_ correlate with brain enzymatic activity of α-, β-, and γ-secretase and the severity of Aβ peptides deposition in the brain.

Despite the significant increases of CSF clusterin and APL1β28 in AD and MCI patients, the average levels of [APL1β28]_plasma_ and [clusterin]_plasma_ were not different in the aMCI subjects compared to those in the healthy controls, a result that is consistent with a previous study by Thambisetty *et al.*^73^. We found that aMCI patients showed an increased trend in clusterin levels and trended toward a decreasing performance in cognitive tests, changes that are identified as potential candidates for early events in the pre-dementia stages. Interestingly, we found that the average levels of [APL1β28]_plasma_ and [clusterin]_plasma_ were not different in the ApoE ε4-carriers and non-carriers. Similar to ApoE, clusterin targets at amyloid-β aggregation and clearance, lipid homeostasis which is related to the pathogenesis of AD^65^,^66^,^67^,^68^,^69^. However, the relationship between ApoE ε4 subtypes and brain clusterin levels remains elusive, which might be due to the complication of this disease^95^. Furthermore, whether the plasma clusterin level reflects the brain clusterin level needs further investigation. In our current study, we did not have enough aMCI-AD conversion cases due to the limited number of aMCI patients and a relatively short monitoring period post-diagnosis. Follow-up cohort studies are needed to evaluate whether the plasma levels of APL1β28 and clusterin could serve as indicators for the conversion rate of aMCI to AD in a Chinese population.

## Materials and Methods

### Ethics statement

All experiments were conducted in accordance with protocols approved by the institutional review board of Peking University Institute of Mental Health. Legally binding informed consent was obtained from all subjects.

### Subjects

All study participants were mainland China citizens of Han ethnicity who were prospectively recruited to establish a case registry at the Dementia Care and Research Center, Peking University Institute of Mental Health. The study, which was conducted from 2006 to 2011, included 47 individuals with amnestic mild cognitive impairment (the aMCI group) and 35 healthy adult controls (the NC group). All the subjects received a clinical review and a battery of neuropsychological tests and laboratory tests; standing body weight was measured with an electronic scale, height (without shoes) was measured with an anthropometer, and the BMI was computed based on these parameters (kg weight/m^2^height).The education level was equated to the amount of time the participant spent in school (elementary school through graduate school).

### Diagnostic criteria

The clinical diagnosis of amnestic MCI was based on the criteria established by Petersen^96^. The diagnostic criteria of aMCI were defined as follows: (1) age ≥ 50 years and met the Petersen criteria; (2) complained of memory impairment that (preferably) could be corroborated by another individual; (3) the impaired memory function was not compatible with patient’s age and/or education; (4) the total MMSE score was no less than 24, and the CDR score was not more than 0.5; (5) the activities of daily living were intact; and (6) DSM-IV criteria for the diagnosis of dementia was not met.

The exclusion criteria were as follows: (1) diagnosed with other neurodegenerative disease; (2) cognitive dysfunction was induced by traumatic brain injuries, tumor or infection; (3) cognitive dysfunction was induced by alcohol or drug use; and (4) cognitive dysfunction was induced for reasons other than mentioned above.

Inclusion criteria for the normal control (NC) group were (1) age ≥ 50 years; (2) no serious physical diseases; and (3) normal cognitive function.

### Neuropsychological assessment

These tests were administered using standardized procedures as previously described^97^,^98^. In brief, the neuropsychological tests were conducted in Chinese by well-trained and certified evaluators. The evaluators were not informed of the subjects’ diagnoses in advance. The MMSE, the MoCA, and the cross-cultural neuropsychological test battery (CCNB), including the Cognitive Ability Screening Instrument (CASI C-2.0), were administered to all participants.

The MMSE, which is widely used as a screening tool to evaluate subjects’ general cognitive function, includes five subtest areas: orientation, registration, recall, attention/concentration/calculation and language; scores range from 0 (severe impairment) to 30 (no impairment)^19^,^99^. Further, general cognitive function was assessed with the MoCA and CASI C-2.0, which provide more comprehensive assessment than the MMSE. The CASI includes nine domains: long-term memory, short-term memory, attention, concentration, orientation, reasoning (comprising abstraction and judgment), language abilities, visual construction and category fluency^100^. The MoCA is designed specifically for patients with MCI or with mild AD. Executive function, short-term memory, language skills and visuo-spatial processing are categories included on the MoCA test.

### Quantitative measurements of APL1β28 and clusterin from human plasma

Plasma APL1β28 and clusterin concentrations were assayed by commercially available ELISA kits (Human APL1β28 ELISA, EK-018–42; Human Clusterin ELISA, EK-018-35, Phoenix Pharmaceuticals, Inc.). All samples were run in duplicate.

### Apolipoprotein (ApoE) genotype

ApoE genotyping (ε2, ε3, and ε4 allele) was performed on DNA samples from the subjects’ blood, as previously described^101^.

### Statistical analyses

Data analysis was performed using IBM SPSS Statistics 16.0 for Windows. We first used partial correlation analysis to investigate the associations between the cognitive tests and plasma clusterin and APL1β28 levels, respectively. Analyses were adjusted for age, sex, ApoE ε4 status and education when applicable. We then compared differences in plasma clusterin and APL1β28 concentrations between the MCI and control groups (independent sample t-tests) to test the entire sample for differences in plasma clusterin and APL1β 28 concentrations and found no significant differences. Finally, within the diagnostic categories, group comparisons of continuous variables according to their ApoE ε4 status were performed using the student’s t-test, which revealed no significant differences. A priori level of significance was set at p<0.05 for all analyses.

## Additional Information

**How to cite this article**: Meng, Y. *et al.* A correlativity study of plasma APL1β28 and clusterin levels with MMSE/MoCA/CASI in aMCI patients. *Sci. Rep.* **5**, 15546; doi: 10.1038/srep15546 (2015).

## Supplementary Material

Supplementary Information

## Figures and Tables

**Table 1:** Demographic Characteristics and Cognitive Performance Scores of Two Subject Groups.

	aMCI (N = 47)	NC (N = 35)	p-value
Age (years)	72.3 (1.0)	69.0 (1.5)	0.062
Sex (M/F)	22/25	13/22	0.381
ApoE ε4 present	27.7%	25.7%	0.844
Education (years)	13.9 (0.5)	14.5 (0.6)	0.386
BMI	23.7 (0.5)^a^	24.5 (0.9)^b^	0.397
MMSE	27.5 (0.2)	28.6 (0.2)	0.002*
MMSE, median	28.0	29.0	–
MoCA	23.0 (0.4)^c^	25.2 (0.4)^d^	0.000*
MoCA, median	23.0	26.0	–
CASI	92.2 (0.8)	95.0 (0.5)	0.007*
CASI, median	93.0	96.0	–

Data are presented as the mean (SD). P-values were obtained using the two-tailed Chi-square test for gender and ApoE ε4; the student’s t-test was used for other factors, *p < 0.01.

^a^Data were missing for nine patients.

^b^Data were missing for one patient.

^c^Data were missing for nine patients.

^d^Data were missing for four patients.

**Table 2:** Correlations of Cognitive Tests with Plasma APL1β28 and Clusterin.

	aMCI Group (N = 47)		NC Group (N = 35)	
	APL1β28	Clusterin	APL1β28	Clusterin
**MMSE, points**				
Unadjusted	0.046*(−0.293)	0.009**(−0.377)	0.871(−0.028)	0.584(−0.096)
Adjusted for age	0.047*(−0.295)	0.013*(−0.363)	0.423(0.142)	0.792(−0.047)
Adjusted for sex	0.059(−0.280)	0.011*(−0.372)	0.496(0.121)	0.886(−0.026)
Adjusted for education	0.055(−0.285)	0.005**(−0.404)	0.484(0.124)	0.808(−0.043)
Adjusted for ApoE ε4	0.053(−0.287)	0.008**(−0.386)	0.416(0.144)	0.834(−0.037)
Adjusted for BMI	0.025*(−0.363)	0.029*(−0.353)	0.466(0.132)	0.934(−0.015)
**MoCA, points**				
Unadjusted	0.677(0.068)	0.012*(−0.392)	0.542(0.114)	0.870(−0.031)
Adjusted for age	0.707(0.062)	0.016*(−0.384)	0.526(0.120)	0.877(−0.030)
Adjusted for sex	0.605(0.085)	0.020*(−0.371)	0.618(0.095)	0.939(−0.015)
Adjusted for education	0.692(0.065)	0.020*(−0.372)	0.481(0.134)	0.966(0.008)
Adjusted for ApoE ε4	0.665(0.071)	0.016*(−0.385)	0.686(0.077)	0.897(−0.025)
Adjusted for BMI	0.489(0.127)	0.025*(−0.396)	0.608(0.100)	0.839(−0.039)
**CASI, points**				
Unadjusted	0.164(−0.206)	0.017*(−0.346)	0.474(−0.125)	0.512(−0.115)
Adjusted for age	0.166(−0.208)	0.025*(−0.031)	0.500(−0.120)	0.508(−0.118)
Adjusted for sex	0.103(−0.244)	0.013*(−0.365)	0.433(−0.139)	0.665(−0.077)
Adjusted for education	0.136(−0.223)	0.021*(−0.340)	0.458(−0.132)	0.546(−0.107)
Adjusted for ApoE ε4	0.161(−0.210)	0.018*(−0.035)	0.318(−0.176)	0.484(−0.124)
Adjusted for BMI	0.188(−0.218)	0.055(−0.314)	0.466(−0.132)	0.583(−0.099)

Data are presented as the p-value (correlation coefficient). P-values were obtained using partial correlation analysis to investigate the correlations between the cognitive test scores and plasma clusterin and APL1β28 levels, respectively. (*p < 0.05, **p < 0.01).
