# Supplementary material for: A correlativity study of plasma APL1β28 and clusterin levels with MMSE/MoCA/CASI in aMCI patients
Source: Sci Rep. 2015 Oct 27;5:15546. doi: 10.1038/srep15546 (PMC4621490; doi:10.1038/srep15546)
Supplement: Supplementary Information [file srep15546-s1.pdf]

Supplemental Information for Meng et al.,

"A correlativity study of plasma APL1β28 and clusterin levels with MMSE/MoCA/CASI in aMCI patients"

Ying Meng[1,2,]*, Huiying Li[1,2,]*, Rui Hua[2]*, Huali Wang[1], Jian Lu[2],

Xin Yu[1,#], Chen Zhang[2,#]

[1]Dementia Care & Research Center, Peking University Institute of Mental Health; Beijing Municipal Key Laboratory for Translational Research on Diagnosis and Treatment of Dementia, National Clinical Research Center for Mental Disorders (Peking University Sixth Hospital);

[2]State Key Laboratory of Membrane Biology, School of Life Sciences; PKU-IDG/McGovern Institute for Brain Research, Peking University, Beijing 100871, China

*These authors contributed equally to this work

# Correspondence should be addressed to Y.X. (yuxin@bjmu.edu.cn), or Z.C. (ch.zhang@pku.edu.cn)

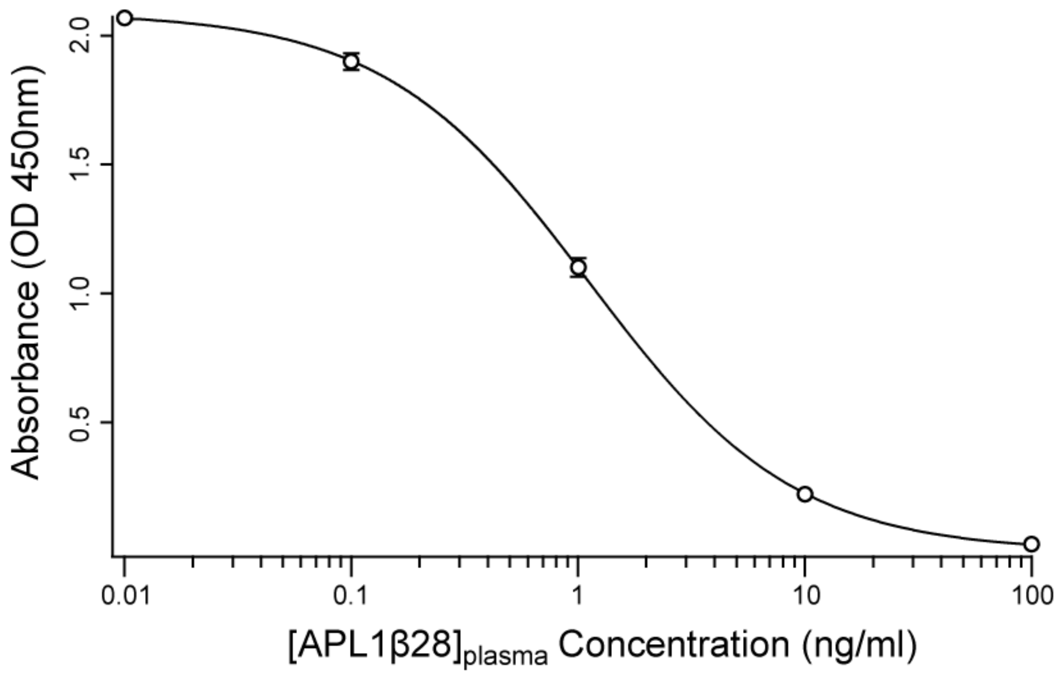

Suppl. Figure 1. The standard curve of human APL1β28 ELISA. The curve results from a non-linear sigmoidal curve fit of the APL1β28 standards. Data points show mean optical density values ± SEM at each concentration.

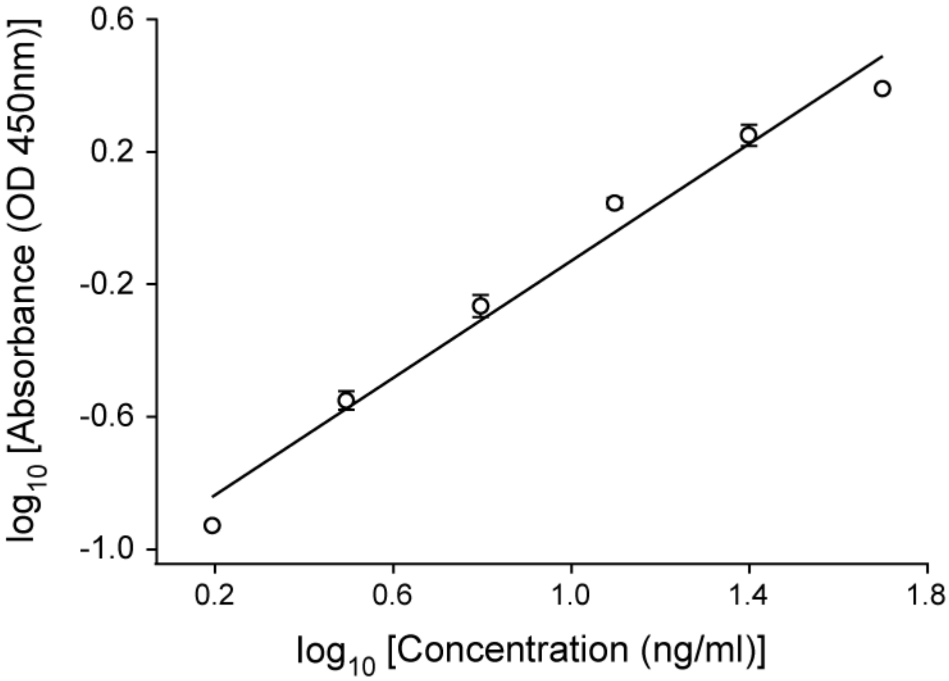

Suppl. Figure 2. The standard curve of human clusterin ELISA. The known concentration of clusterin standard samples and its corresponding optical density reading is plotted on the log scale (X-axis) and on the log scale (Y-axis), respectively. The solid line represents the best fit as determined by linear curve fitting (r^2 = 0.977).

Suppl. Table 1: P-values of a One-sample Kolmogorov-Smirnov Test.

	**APL1β28**	**Clusterin**	**MMSE**	**MoCA**	**CASI**
**aMCI group**	0.163	0.795	0.342	0.516	0.590
**NC group**	0.337	0.284	0.001**	0.085	0.241

$^{**}p < 0.01$
